# Building and Sustaining a Statewide Telepsychiatry Network: Lessons Learned from the North Carolina Statewide Telepsychiatry Program (NC-STeP)

**DOI:** 10.3390/ijerph23040508

**Published:** 2026-04-16

**Authors:** Sy Atezaz Saeed

**Affiliations:** 1 North Carolina Statewide Telepsychiatry Program (NC-STeP), Brody School of Medicine, East Carolina University, Greenville, NC 27834, USA; saeeds@ecu.edu; Tel.: +1-252-744-0454; 2 Center for Telepsychiatry and e-Behavioral Health, Brody School of Medicine, East Carolina University, Greenville, NC 27834, USA; 3 Department of Psychiatry and Behavioral Medicine, Brody School of Medicine, East Carolina University, Greenville, NC 27834, USA

**Keywords:** telepsychiatry, emergency department, boarding, involuntary commitment, cost savings, interoperability, maternal health, pediatrics, university students, health equity

## Abstract

**Highlights:**

**Public health relevance—How does this work relate to a public health issue?**

- North Carolina’s statewide telepsychiatry network (NC-STeP) addresses psychiatric workforce shortages and ED boarding across rural and underserved regions.
- Telepsychiatry enables timely crisis assessment and safe community discharge, improving throughput and access to behavioral health care statewide.

**Public health significance—Why is this work of significance to public health?**

- Over 13 years, NC-STeP reduced unnecessary psychiatric hospitalizations and generated substantial cost avoidance for the health system.
- Program expansions (maternal, pediatric, university settings) demonstrate scalable integration of specialty behavioral health in routine care.

**Public health implications—What are the key implications or messages for practitioners, policy makers and/or researchers in public health?**

- Statewide telepsychiatry can function as core clinical infrastructure when financing, interoperability, and equity measurement are aligned.
- Embedding equity-focused strategies is essential to ensure telehealth expansion does not widen racial or rural access disparities.

**Abstract:**

Background: North Carolina faces persistent shortages of psychiatric professionals, particularly in rural and underserved regions, resulting in prolonged emergency department (ED) boarding, avoidable psychiatric hospitalizations, and inequitable access to behavioral health services. The North Carolina Statewide Telepsychiatry Program (NC-STeP), launched in 2013, is one of the nation’s longest-running statewide telepsychiatry programs. Objective: To summarize the development, implementation, outcomes, and lessons learned from NC-STeP across ED, community, maternal, pediatric, and university settings. Methods: Data were synthesized from NC-STeP operations, service data, and peer-reviewed publications (2013–2025). Results: NC-STeP completed 67,543 ED psychiatric assessments, prevented 11,802 hospitalizations, and generated $63.7 million in cost savings. Telepsychiatry increased safe discharges, reduced ED boarding, improved access, and revealed persistent equity gaps. Conclusions: NC-STeP demonstrates a scalable statewide telepsychiatry model improving throughput, reducing avoidable admissions, and expanding equitable behavioral health access.

## 1. Introduction

Mental disorders are among the leading causes of morbidity, disability, and premature mortality worldwide. In the United States, approximately 26.2% of adults experience a mental disorder in any given year [1]. Despite the prevalence of mental illness, nearly half of adults with a mental health condition and 80% of individuals needing substance use treatment do not receive needed care [2].

In North Carolina, these access challenges are intensified. More than 54% of adults with mental illness receive no treatment [2], over 70% of children with treatable mental disorders remain untreated [3], and 93 counties qualify as Mental Health Professional Shortage Areas [4]. Workforce maldistribution is particularly severe in rural counties, which frequently lack psychiatrists altogether [5]. Consequently, emergency departments (EDs) have become de facto mental health providers. In 2013, NC hospitals had 162,000 behavioral health ED visits. From 2008 to 2010, in North Carolina, 10% of ED visits had one or more mental health diagnosis (MHD) code assigned to visit; twice the estimated national average [6]. There was a 17.7% increase in rate of ED visits of patients with MHD; compared to 5.1% increase in overall rate of ED visits and people with mental health disorders were admitted to the hospital at twice the rate of those without [6].

Individuals with psychiatric crises often experience prolonged ED boarding times, delayed treatment, and unnecessary hospitalization often due to limited psychiatric expertise onsite. Telepsychiatry has emerged as a viable solution to these systemic challenges. Prior studies demonstrate that telepsychiatry increases access, reduces geographic disparities, enhances continuity of care, and lowers healthcare costs [7]. The North Carolina Statewide Telepsychiatry Program (NC-STeP) was designed to address these barriers through a coordinated, technology-enabled psychiatric service delivered across diverse clinical settings [8]. Established under North Carolina Session Law 2013-360 and launched in 2013, NC-STeP began with 28 hospitals, ultimately expanding to 76 ED sites. The program’s vision was to ensure that anyone presenting with a behavioral health crisis receives timely psychiatric assessment and linkage to appropriate care. After its initial success, in 2018, the North Carolina Legislature expanded NC-STeP to include community-based primary care clinics, significantly broadening its reach beyond EDs.

Multiple NC-STeP research studies, as well as clinical programs, have evaluated its impact on access to care, ED workflow, patient outcomes, cost reductions, telepsychiatry utilization patterns, and equity. This paper summarizes lessons learned from the last 13 years of successful operation of NC-STeP and synthesizes findings from multiple NC-STeP program evaluations to identify cross-cutting lessons relevant to statewide telepsychiatry implementation. Rather than a pure narrative review, or a single, hypothesis-driven program evaluation, this paper provides a multi-domain program evaluation synthesis with narrative integration and lessons learned.

### Conceptual Model: NC-STeP as a Statewide Telepsychiatry Platform

NC-STeP is a hub-and-spoke statewide telepsychiatry platform that delivers psychiatric expertise across settings, populations, and levels of acuity using shared infrastructure, workforce, workflows, and governance. The key features of the model include a central hub, clinical spokes (care settings), and a bidirectional flow.

**Central Hub components include:**

- Statewide telepsychiatry network (NC-STeP);
- Shared psychiatric workforce;
- Unified technology platform and interoperability layer;
- Common clinical standards, measurement-based care, and equity monitoring;
- Centralized scheduling, credentialing, and quality oversight.

**Clinical “Spokes” (Care Settings) include:**

- Emergency Departments (high-acuity crisis care);
- Maternal Health (perinatal psychiatry and collaborative care);
- Adult Primary Care (early identification and treatment);
- Pediatric Primary Care (early identification and treatment);
- University Health Centers (young adult mental health & prevention).

**Bidirectional Flow includes:**

- Sites access psychiatric expertise via telepsychiatry;
- Clinical data and outcomes flow back to the hub;
- Shared learning improves system-wide performance and equity.

**Figure 1:**
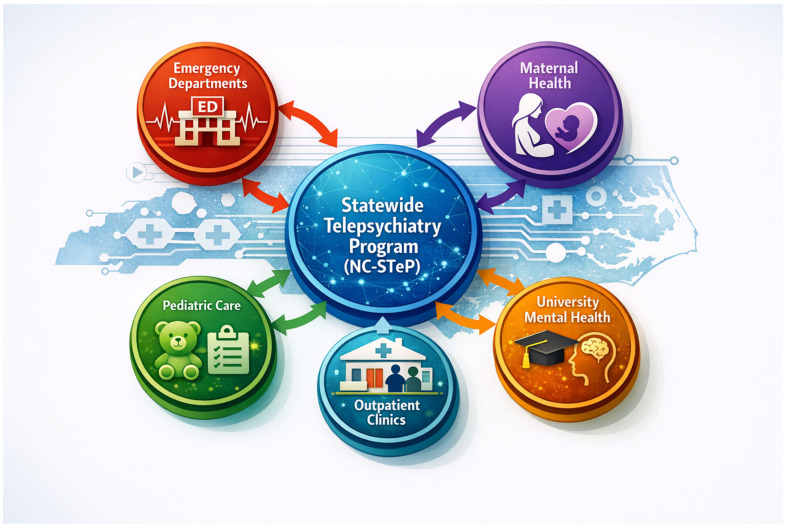
Conceptual Model of the North Carolina Statewide Telepsychiatry Program (NC-STeP) Integrating Emergency, Outpatient, Maternal, Pediatric, and University Mental Health Services. Figure 1 was generated using Microsoft Copilot (2026.408.2119.0).

Figure 1 below illustrates the North Carolina Statewide Telepsychiatry Program (NC-STeP) as a centralized statewide behavioral health platform.

NC-STeP functions as an integrated statewide platform rather than a series of independent telepsychiatry initiatives. As shown in Figure 1, emergency, maternal, pediatric, and university programs are unified through a centralized telepsychiatry hub that provides shared workforce, technology, and governance. This hub-and-spoke structure allows psychiatric expertise to be deployed flexibly across care settings while maintaining consistent clinical standards, interoperable workflows, and equity monitoring. The model enables population-specific adaptations without fragmenting infrastructure, supporting scalability and sustainability at the state level.

## 2. Materials and Methods

### 2.1. Study Design

This paper synthesizes NC-STeP program data, peer-reviewed studies, operational evaluations, and system-level analytics from 2013–2025.

### 2.2. Data Sources

- NC-STeP clinical operations data;
- Program evaluation dashboards;
- Peer-reviewed publications;
- COVID-19 telepsychiatry utilization data;
- Hospital ED operational metrics.

### 2.3. Data Analysis

The following data were analyzed for this paper:

- ED length of stay;
- Hospitalization outcomes;
- Cost-savings estimates;
- Telepsychiatry utilization differences by race/sex;
- Program expansions into maternal, pediatric, and university settings.

### 2.4. Ethical Considerations

This study involved secondary analysis of de-identified program data. Ethical review and approval were not required for this study because it involved secondary analysis of de-identified program data and did not meet the definition of human subjects’ research.

## 3. Results (Lessons Learned Across Core Program Domains)

### 3.1. Electronic Health Records Technology and Workflow

Telepsychiatry is a viable option for providing psychiatric care to those who are currently underserved or who lack access to services. While the current technology is adequate for most uses, and continues to advance, there remain barriers to its widespread utilization. One such barrier when working with different healthcare systems is that they utilize different electronic health record systems (EHRs). For a successful clinical encounter, a provider needs to see a patient as well as review his or her health record. That is true whether the clinical encounter takes place in-person or via telepsychiatry. However, obtaining access in real time to patient’s health records, including lab results and other pertinent clinical information, may not be straightforward if the provider does not have the ability to view the patient’s electronic health record. This is often the case when the patient and provider are in different health care systems that use different EHRs.

At the time NC-STeP went live, a fully functional health information exchange (HIE) was not available in many states, including North Carolina. To meet its needs, the program envisioned a telepsychiatry portal that supported scheduling, clinical data exchange, and encounter reporting across the network. NC-STeP developed an integrated portal enabling ED physicians to request consults, staff to input patient data, telepsychiatrists to conduct evaluations, and ED physicians to make disposition decisions.

Given the heterogeneity of EHRs across 108 hospitals across the state of North Carolina, NC-STeP implemented interoperability solutions using Direct Messaging and C-CDA standards. The portal provides a single platform for telepsychiatry assessments across EDs and providers regardless of EHR vendor. NC-STeP’s EHR interoperability experience has been described in prior publications [9,10]. Key technology and workflow lessons learned are summarized in Table 1.

### 3.2. Emergency Department Boarding Study

The number of patients seeking treatment in emergency departments (EDs) for mental health reasons continues to rise, contributing to ED overcrowding and prolonged psychiatric patient boarding [11,12]. Telepsychiatry has been proposed as a solution to provide timely psychiatric consultation to improve patient flow and disposition.

In 2020, NC-STeP published findings from a large retrospective observational study comparing ED length of stay (LOS) and disposition during NC-STeP active versus inactive periods [13]. Extended LOS (>2 days) was used as a proxy for psychiatric boarding.

Results showed higher home discharge rates during active periods and lower psychiatric transfers among patients boarded for >2 days. Telepsychiatry had the strongest impact in reducing psychiatric transfers for extended length of stay patients (>2 days). This group reflects true psychiatric boarding. The results for this group when NC-STeP was active showed that 61.7% patients were discharged home (vs. 42.8% when inactive) and 29.3% were transferred to psychiatric hospitals (vs. 46.2% when inactive). Overall and LOS-stratified ED disposition outcomes are presented in Table 2.

### 3.3. Cost-Savings Study

Emergency departments have experienced a substantial rise in visits related to mental health and substance use disorders (M/SUDs) [14]. This surge has intensified challenges such as overcrowding, resource limitations, and uneven access to psychiatric expertise. Because community-based behavioral health services are often insufficient or inaccessible, EDs have increasingly become default providers of psychiatric care, especially for uninsured and low-income individuals protected under Emergency Medical Treatment and Labor Act (EMTALA), a 1986 federal law requiring Medicare-participating hospitals with emergency departments to provide a medical screening exam and stabilizing treatment for anyone with an emergency medical condition, regardless of ability to pay and prohibits transferring patients for financial reasons before they are stabilized. Many psychiatric emergencies can be resolved in ED with proper evaluation and treatment. However, many ED physicians lack specialized training in behavioral health and may “overprescribe” hospitalization recommendations to be safe, which can contribute to unnecessary psychiatric hospitalizations—including involuntary commitments (IVCs). ED-based telepsychiatry is one possible solution. Telepsychiatry consultation services in the EDs can decrease unnecessary psychiatric hospitalizations and contribute to significant cost savings. Avoiding unnecessary psychiatric hospitalization can promote patient satisfaction, reduce costs, and improve outcomes for the patients and families. Prompt, specialized evaluation can often resolve psychiatric emergencies without hospitalization.

A NC-STeP cost-savings study covering November 2013–June 2020 analyzed 19,383 ED encounters involving telepsychiatry consultation [15]. Using North Carolina’s standardized reimbursement rate for state-funded “three-way” psychiatric beds [16], the study estimated cost savings associated with avoided hospitalizations.

Over a 6.5-year period, NC-STeP generated more than $20 million in estimated cost savings by overturning 4627 inappropriate IVCs. Cost-savings metrics and utilization outcomes are summarized in Table 3.

### 3.4. Equitable Access to Care Through Telepsychiatry- COVID-19 Utilization Study

The COVID-19 pandemic increased behavioral health needs and accelerated telepsychiatry adoption nationwide. NC-STeP evaluated telepsychiatry utilization across 27 NC EDs before, during, and after the COVID-19 lockdown [17].

Telepsychiatry use increased sharply during lockdown and demonstrated high scalability and resilience. However, utilization increases were uneven across demographic groups, with larger increases among White patients and women compared with Black patients and men. These findings highlight persistent equity gaps in emergency mental health care. Key utilization and demographic patterns are summarized in Table 4.

In summary, the pandemic increased behavioral health needs and telepsychiatry use but also revealed persistent inequities. Telepsychiatry played a key role in stabilizing ED psychiatric care, and achieving equitable access will require sustained, intentional efforts. These disparities emphasize that technology alone cannot guarantee equitable care; rather, telepsychiatry programs must be paired with intentional policies aimed at addressing referral biases, strengthening trust and engagement in underserved communities, and ensuring that emergency pathways do not inadvertently perpetuate inequities. As states continue integrating telepsychiatry into routine emergency care, these findings point to the need for targeted outreach, culturally responsive practices, and ongoing monitoring of disparities to ensure that telehealth expansion advances—not undermines—health equity. Ultimately, the pandemic provided a natural stress test for statewide telepsychiatry infrastructure such as NC-STeP, demonstrating both its capacity to absorb system shocks and the importance of embedding equity-focused strategies into its future evolution.

### 3.5. MOTHeRS (Maternal Outreach Using Telehealth for Rural Sites) Program

The COVID-19 pandemic worsened long-standing maternal health disparities in Eastern North Carolina, a region marked by high rates of poverty, chronic illness, food insecurity, and limited access to medical and mental health services. These challenges contribute to disproportionately high maternal and infant mortality rates—especially among Black, American Indian/Alaska Native, low-income, and rural women.

To address these issues, NC-STeP expanded its statewide telepsychiatry network to create the MOTHeRS Project (Maternal Outreach Through Telehealth for Rural Sites). Beginning in 2020, the program integrated comprehensive maternal–fetal and behavioral health services directly into rural obstetric clinics through telehealth. A multidisciplinary team—including maternal–fetal medicine specialists, psychiatrists, behavioral health managers, dietitians, diabetes educators, and nurse navigators—works alongside local OB-GYN providers to deliver coordinated, patient-centered care.

The project also screened all patients for depression, anxiety, and food insecurity. Those in need received rapid behavioral health consultation, mental health treatment via telepsychiatry, and medically tailored food support with nutrition education. Remote ultrasound review and specialty consultations reduced travel burdens for high-risk pregnancies. The program delivered coordinated telehealth services across four rural counties, improving access to high-risk pregnancy care and perinatal mental health services [18].

The project reduced travel burden, expanded behavioral health access, and screened extensively for food insecurity. Quantitative outcomes across maternal–fetal care, mental health visits, travel savings, and food support are summarized in Table 5.

The MOTHeRS Project demonstrates a scalable, effective approach to reducing rural maternal health disparities by integrating specialty care, behavioral health, and social support through telehealth. As maternal mortality continues to rise—especially among minority and rural populations—policy action to expand such models is urgent. Strengthened investment in telehealth-enabled collaborative care can ensure safer pregnancies and healthier outcomes for mothers and infants across rural America.

### 3.6. Impact of Education Strategies on Individuals’ Attitude Towards Telemental Health Service

Nearly half of individuals with significant mental health symptoms still do not seek care due to barriers such as stigma, cost, limited trust in the mental health system, lack of mental health providers, and uncertainty about where to find help [2,19]. Telemental health offers a promising solution by expanding access, reducing costs, and increasing convenience—but adoption remains limited.

A survey experiment evaluated the impact of peer-narrated versus professional-narrated educational videos on attitudes toward telemental health use [20]. Peer-narrated messaging influenced a broader range of attitudinal constructs and led to deeper information processing, although overall intention to use services was similar across groups.

This study examined whether different educational strategies could improve individuals’ attitudes toward and intentions to use telemental health services. Drawing on multiple theories—including the AIDA (Attention, Interest, Desire, and Action) model, technology acceptance model (TAM), theory of reasoned action (TRA), heuristic–systematic processing, and social identity theory—the researchers compared two educational videos: one narrated by a peer (in-group) and one by a professional (out-group). A survey experiment with 282 students at one of the rural HBCUs (Historically Black Colleges and Universities) found that:

- Attitude strongly predicts intention to use telemental health services.
- The peer-narrated video influenced a wider range of factors shaping attitudes, including ease of use, subjective norms, trust, relative advantage, and stigma.
- The professional-narrated video influenced only trust and relative advantage.
- Overall attitude and intention levels were similar across groups, but peer-based messaging led to deeper, more comprehensive information processing.

These findings suggest that in-group, peer-driven education strategies may help individuals consider more dimensions of telemental health—particularly social factors like stigma—leading to more informed and nuanced attitudes. This has important implications for designing culturally relevant educational materials that address disparities in mental health service use, especially among underserved racial minority populations.

### 3.7. Pediatric Mental Health Initiative (NC-STeP-Peds)

The NC-STeP-Peds initiative expanded the North Carolina Statewide Telepsychiatry Program (NC-STeP) to meet the rising mental health needs of children and adolescents, especially those living in rural and underserved areas through a $3.2 million, three-year grant funding from the United Health Foundation.

The NC-STeP-Peds model delivers pediatric mental health care through a comprehensive, team-based telepsychiatry approach. Core elements include pediatrician-led care supported by mental health clinicians and psychiatric consultants, evidence-based and measurement-based treatment, universal behavioral health screening, family engagement, and collaboration with schools and community agencies. Key project components include embedding licensed behavioral health providers in six practices, delivering psychiatric consultation via telemedicine, building a virtual-reality community (“NC Rural Kids Get Well”) for education and peer support, and developing an AI-driven knowledge management portal to support collaboration and family engagement. The program also serves as a training platform and collaborates with community and university partners to build a broader continuum of care. Telepsychiatry serves as the primary modality, aligning well with children’s comfort with technology. The clinical workflow begins with universal screening in pediatric practices, followed by referrals, scheduled telepsychiatry sessions, and virtual psychiatric evaluations. Pediatricians manage medications, while behavioral health providers offer therapy, maintain patient registries, coordinate care, and meet regularly with child psychiatrists.

Program outcomes are evaluated across four domains:

- Access: wait times, patient volumes, follow-ups, re-consults, and underserved population metrics;
- Effectiveness: improvements on validated measures;
- Patient Experience: satisfaction and visit adherence;
- Continuity: follow-up consistency and visit frequency.

As of January 2026, the program screened 43,858 children, completed over 700 psychiatric evaluations, and delivered 1858 mental health visits.

These outcomes demonstrate improved early identification and access to pediatric behavioral health services in underserved areas. Program activities and outcomes are summarized in Table 6.

### 3.8. University Students’ Mental Health Initiative

Mental health problems are common among college students. Although university students report levels of mental health similar to their non-university counterparts [21], recent studies suggest an increase and severity of mental problems and help-seeking behaviors in university students around the world in the last decade [22]. Some researchers refer to these trends as an emerging “mental health crisis” in higher education [23]. Despite this burden, many students—especially those from minority backgrounds living in rural areas—face reduced access to mental health services and cultural or normative barriers (e.g., stigma) that suppress help-seeking and exacerbate disparities. There are many adverse outcomes associated with psychological distress in early adulthood. Adverse short-term outcomes include poor college attendance, performance, engagement, and completion [24]. Long-term adverse outcomes include dysfunctional relationship [25], recurrent mental health problems, university dropout, lower rates of employment, and reduced personal income [26].

Against this backdrop, NC-STeP launched, in spring 2022, a five-year initiative at Elizabeth City State University (ECSU), an HBCU in Pasquotank County, with investment from Blue Cross and Blue Shield Foundation of North Carolina. The partnership sought to address local psychiatric workforce shortages and strengthen campus connectedness as mental health needs surged.

Operationally, the model emphasizes universal screening for mental health conditions; systematic assessment and monitoring using validated scales (e.g., PHQ-9, GAD-7); joint care planning with care plan revision if not improving; facilitation and coordination of behavioral health treatment; and continuity with an appointed care team member. The integrated care team typically includes the primary care provider (PCP), behavioral health manager (care manager/therapist), and a psychiatric consultant, with workflows designed so psychiatrists collaborate with a care manager while the PCP remains the longitudinal prescriber.

Students enter via multiple routes: (1) referral from the campus PCP following elevated PHQ-9 or GAD-7 scores or observed concerns; (2) self-presentation to health services requesting counseling; or (3) referral from the counseling center. Importantly, NC-STeP consults occur within the student health center, reducing segregation between physical and mental health services.

The on-campus team (psychiatric NPs/psychiatrists and counseling staff) co-manages care and executes ongoing outreach: residence-hall and classroom education, athletic team activities, “Viking Visits” in academic buildings, and frequent tabling at orientations, open houses, dining halls, games, and community events.

From 2022–2025, the initiative completed thousands of screenings, facilitated referrals, and delivered hundreds of psychiatric and counseling encounters. Program utilization and outcomes are summarized in Table 7.

The NC-STeP’s Students’ Mental Health Initiative demonstrates a scalable, equitable model that:

- Reduces treatment gaps;
- Increases early detection;
- Improves student engagement and outcomes;
- Overcomes provider shortages;
- Supports minority and rural student populations.

The NC-STeP model demonstrates that telepsychiatry is a practical solution to campus mental health workforce shortages, suggesting that it may be worthwhile to mandate or incentivize telepsychiatry integration within student health systems at public universities, especially rural campuses and HBCUs.

## 4. Discussion

The North Carolina Statewide Telepsychiatry Program (NC-STeP) demonstrates that a coordinated, statewide telepsychiatry network can function as durable clinical infrastructure rather than a stop-gap solution to psychiatric workforce shortages. Over more than a decade of operation, NC-STeP has shown that telepsychiatry can be integrated across heterogeneous health systems, expand access in rural and underserved communities, improve emergency department (ED) throughput, and support equitable delivery of behavioral health care across the life course.

### 4.1. System-Level Impact and ED Performance

NC-STeP’s most direct system-level impact has been its ability to improve ED management of psychiatric crises. Hospitals participating in the program consistently report reductions in ED boarding, faster access to psychiatric consultation, and streamlined disposition processes [27]. As summarized in the Results and accompanying tables, telepsychiatry use was associated with improved disposition decisions, particularly among patients experiencing prolonged ED boarding. Rather than restating specific metrics, the key lesson is that timely access to psychiatric expertise—regardless of hospital size or location—changes clinician behavior, reduces defensive hospitalization practices, and enables safe discharge planning even in resource-constrained settings.

For rural and small hospitals, participation in a statewide telepsychiatry network effectively substitutes for on-site psychiatric staffing that would otherwise be not feasible [28]. This finding has important implications for states grappling with uneven psychiatric workforce distribution and growing ED overcrowding.

### 4.2. Financial Sustainability and Policy Relevance

Beyond clinical outcomes, NC-STeP highlights the economic case for telepsychiatry as a public health investment. Avoidance of unnecessary psychiatric hospitalizations—particularly involuntary commitments—represents not only direct cost savings but also reduced strain on state psychiatric facilities, emergency medical services, and law enforcement. These downstream effects are rarely captured in traditional cost analyses yet are central to health system sustainability.

Importantly, NC-STeP’s cost savings were achieved without relying on speculative national cost estimates, instead using state-standardized reimbursement approaches. This strengthens the program’s relevance for policymakers and supports the argument that telepsychiatry is fiscally responsible when aligned with appropriate payment models.

### 4.3. Technology as an Enabler, Not a Barrier

A key lesson from NC-STeP is that technology barriers—particularly fragmented electronic health record (EHR) systems—can be overcome without comprehensive system replacement. The program’s interoperability strategy illustrates that workflow-driven design, rather than technology-driven mandates, enables scalability. By prioritizing seamless clinical exchange over uniform platforms, NC-STeP avoided a common failure mode of large telehealth initiatives.

This experience suggests that states seeking to replicate telepsychiatry networks should focus on interoperability standards, flexible portals, and clinical usability rather than attempting statewide EHR consolidation [29].

### 4.4. Equity Considerations and the Limits of Technology

While NC-STeP expanded access overall, utilization patterns during the COVID-19 pandemic revealed persistent racial and sex-based disparities. These findings reinforce a critical point: telehealth does not inherently produce equity [30,31]. Instead, telepsychiatry reflects—and may amplify—existing structural inequities unless intentionally designed to address them.

The implications are clear. Statewide telepsychiatry programs must incorporate equity monitoring, culturally responsive workflows, unbiased referral pathways, and targeted community engagement. Equity should be treated as a measurable system outcome, not an assumed byproduct of technological expansion.

### 4.5. Scalability Across the Care Continuum

One of NC-STeP’s distinguishing features is its successful expansion beyond EDs into maternal health, primary care, pediatrics, and university settings. These initiatives demonstrate that telepsychiatry can be adapted to diverse clinical environments while maintaining core principles: team-based care, measurement-based practice, and integration into existing workflows.

Rather than viewing these expansions as separate programs, they illustrate a unifying statewide platform capable of supporting population-specific adaptations. This scalability is particularly relevant for states seeking to address behavioral health needs across multiple populations without building parallel systems.

### 4.6. Implications for Replication and Policy

The NC-STeP experience suggests that statewide telepsychiatry efforts are most effective when three conditions are met:

(1) financing aligns with collaborative and consultative care models;
(2) interoperability is operationalized through practical, standards-based solutions; and
(3) equity is explicitly measured, monitored, and managed.

For policymakers, this implies the need for reimbursement parity, streamlined licensure and credentialing, and accountability structures that include equity metrics. For health systems, it underscores the value of central coordination, shared infrastructure, and data-driven workforce planning.

### 4.7. Strengths, Limitations, and Generalizability

#### 4.7.1. Study Limitations

This paper synthesizes findings from operational data, observational studies, and published evaluations of the North Carolina Statewide Telepsychiatry Program (NC-STeP) over more than a decade. As such, several limitations should be considered when interpreting the results.

First, much of the evidence summarized derives from observational and retrospective analyses, rather than randomized controlled trials. Although these designs are well suited to evaluating real-world system-level interventions, they limit the ability to infer causal relationships between telepsychiatry implementation and outcomes such as emergency department (ED) throughput, disposition, and cost savings. Differences observed between program-active and inactive periods may partially reflect unmeasured confounders, including local staffing changes, hospital policies, or other broader trends in behavioral health care.

Second, the cost-savings estimates focus primarily on avoided psychiatric hospitalizations, particularly overturned involuntary commitments, and do not capture the full spectrum of economic effects. Downstream benefits—such as impact on ED throughput, reductions in law enforcement involvement, ambulance transport, caregiver burden, or long-term health outcomes—were not quantified. Conversely, start-up costs, technology maintenance, and workforce training investments were not fully incorporated into the estimates, which may lead to either under- or overestimation of net savings.

Third, program utilization and equity analyses rely on administrative and clinical operational data, which may be subject to data completeness, coding variability across sites, and limited availability of social determinants of health variables. While race and sex differences in utilization were examined, other important dimensions of equity—such as language, disability status, insurance churn, or immigration status—could not be consistently assessed across participating sites.

Fourth, although NC-STeP expanded into maternal, pediatric, and university settings, outcome evaluation for these initiatives is less mature than for the ED-based program. Some expansions were supported by time-limited grant funding, and longer-term sustainability and outcome trajectories are still emerging. As a result, conclusions regarding these models should be interpreted as promising but preliminary.

Finally, NC-STeP benefits from strong academic leadership, long-standing legislative support, and deep partnerships with state agencies. These contextual factors may not be immediately replicable in all jurisdictions and should be considered when interpreting program success.

#### 4.7.2. Generalizability to Other States

Despite these limitations, the findings from NC-STeP have high relevance and transferability to other states facing similar behavioral health workforce shortages, ED boarding challenges, and rural access disparities. While NC-STeP operates within North Carolina’s specific regulatory and financing environment, several core elements of the model are structurally generalizable.

First, psychiatric workforce maldistribution, ED psychiatric boarding, and reliance on EDs as safety-net providers are national challenges, particularly in rural and underserved regions. States with similar demographic patterns, hospital networks, and shortages—especially in the Southeast, Midwest, and Mountain West—are likely to encounter comparable care gaps that telepsychiatry can address.

Second, NC-STeP’s interoperability-focused technology strategy enhances generalizability. Rather than relying on a single electronic health record or proprietary platform, the program demonstrated that standards-based tools (e.g., Direct Messaging and C-CDA) can support statewide telepsychiatry across heterogeneous health systems. This approach is applicable to states with fragmented hospital ownership and varied EHR vendors.

Third, the program’s financing logic—using telepsychiatry to reduce inappropriate hospitalizations and improve ED throughput—aligns with value-based care, Medicaid cost containment, and public hospital sustainability goals shared by most states. Although specific reimbursement mechanisms differ, the underlying economic rationale is broadly applicable, particularly for states bearing high costs of involuntary commitments and state-funded psychiatric beds.

Fourth, NC-STeP’s expansions into primary care, maternal health, pediatrics, and university settings illustrate a platform model rather than siloed programs. This adaptability across populations supports generalizability to states seeking to build integrated behavioral health infrastructure that serves multiple priority groups while leveraging a shared workforce and technology base.

At the same time, successful replication elsewhere will require adaptation rather than direct transplantation. Differences in Medicaid policy, telehealth reimbursement, licensure compacts, hospital governance, and broadband availability will shape implementation. Importantly, NC-STeP’s experience underscores that telepsychiatry alone does not guarantee equitable access; equity-focused referral practices, culturally responsive care models, and real-time monitoring of utilization disparities must be deliberately embedded in any statewide effort.

In summary, while NC-STeP reflects North Carolina-specific history and policy context, the program provides a replicable blueprint for other states. Its central lessons—regarding governance, interoperability, workforce extension, and equity-conscious design—are highly generalizable, provided that local regulatory, financing, and community contexts are explicitly addressed.

## 5. Conclusions

Psychiatric workforce shortages continue to worsen nationwide, with the most severe impacts concentrated in rural and underserved communities. These shortages place sustained pressure on emergency departments, delay access to timely behavioral health care, and contribute to avoidable hospitalizations and poorer patient outcomes. Against this backdrop, the North Carolina Statewide Telepsychiatry Program (NC-STeP) demonstrates that statewide telepsychiatry can function as a scalable, cost-effective model for delivering high-quality psychiatric care through telehealth.

Over more than thirteen years of operation, NC-STeP has shown that coordinated telepsychiatry meaningfully improves system performance by reducing emergency department boarding, increasing the timeliness of psychiatric evaluations, and preventing unnecessary psychiatric hospitalizations. The program’s ability to integrate seamlessly across diverse clinical environments highlights the flexibility required to address behavioral health workforce gaps at scale and supports telepsychiatry’s role as durable clinical infrastructure rather than a temporary workforce substitute.

NC-STeP’s expansion beyond emergency departments into primary care, maternal health, pediatric care, and university health settings further demonstrates telepsychiatry’s capacity to extend psychiatric expertise across the life course. In maternal–fetal medicine, telepsychiatry supports high-risk pregnancies and reduces travel burdens; in primary care and pediatric practices, it strengthens early identification and treatment of behavioral health needs; and in university health centers, it improves access to care for college students facing rising mental health demands. Embedding psychiatric expertise within routine care settings reduces geographic and structural barriers while strengthening continuity and early intervention, underscoring telepsychiatry’s role as a foundational component of an equitable behavioral health system.

The NC-STeP experience indicates that telepsychiatry is most effective as core clinical infrastructure when three conditions are met: financing aligns with collaborative-care workflows; interoperability supports streamlined, cross-system information exchange; and equity is actively measured and managed using real-time, stratified data with targeted outreach. When these conditions are in place, statewide telepsychiatry can reliably reduce boarding, avoid unnecessary hospitalization, and expand equitable access to behavioral health care across settings and populations.

From a policy perspective, statewide telepsychiatry programs warrant recognition as essential behavioral health infrastructure rather than supplemental services. Policymakers can accelerate impact by adopting permanent reimbursement parity for telepsychiatry within Medicaid and commercial plans, aligning payment models with avoided hospitalizations and emergency department throughput gains, and reducing administrative barriers through streamlined licensure and credentialing mechanisms. Requiring routine, stratified equity reporting and linking funding to demonstrated reductions in racial, geographic, and rural disparities are critical to ensuring that telehealth expansion advances equity rather than reinforcing existing gaps. Treating telepsychiatry as a public health and emergency preparedness asset—analogous to trauma systems or poison control networks—can ensure continuity of psychiatric care during workforce shortages, public health emergencies, and future system shocks.

Beyond policy reforms, successful statewide telepsychiatry also requires sustained attention to clinical and system-level implementation. Health systems should standardize workflows, embed measurement-based care, and ensure closed-loop referrals across emergency and outpatient settings to improve quality and continuity. Workforce sustainability depends on integrating telepsychiatry training into clinical education, supporting team-based collaborative care, and reducing professional isolation in rural sites. Equity-centered program design—grounded in community partnerships, culturally responsive care, and routine auditing of referral pathways—is essential to ensuring that telepsychiatry benefits are broadly and fairly distributed. Ongoing evaluation using implementation science and real-time operational dashboards can support continuous learning and adaptation, translating telepsychiatry into durable public health impact.

## Figures and Tables

**Table 1:** Key Lessons Learned from Electronic Health Records (EHR) Technology and Workflow Integration.

Domain	Challenge	NC-STeP Approach	Key Lesson Learned
Interoperability	Multiple EHR vendors across hospitals	Vendor-agnostic integrated portal	Statewide telepsychiatry is feasible without EHR standardization
Real-time Access	Limited access to patient records during consults	Secure portal with Direct Messaging	Real-time clinical decision-making improved
Health Information Exchange	Absence of mature statewide HIE when NC-STeP started	Use of Direct Messaging and C-CDA	Functional HIE can be achieved using national standards
Workflow Coordination	Fragmented scheduling and documentation	Unified telepsychiatry portal	Improved efficiency across EDs and providers
Scalability	Growth to >100 hospitals and outpatient clinics	Centralized, scalable platform	Telepsychiatry can scale statewide with appropriate infrastructure

Abbreviations: EHR, electronic health record; ED, emergency department; HIE, health information exchange; C-CDA, Consolidated Clinical Document Architecture.

**Table 2:** Emergency Department Patient Disposition during NC-STeP Active vs. Inactive Periods (2012–2017).

Outcome Measure	NC-STeP Active	NC-STeP Inactive
Total ED visits (N)	44,857	42,074
Discharged home (%)	76.0	72.2
Psychiatric hospital transfer (%)	16.4	16.0
LOS > 2 days—discharged home (%)	61.7	42.8
LOS > 2 days—psychiatric transfer (%)	29.3	46.2

*LOS > 2 days was used as a proxy for psychiatric boarding. LOS, length of stay; ED, emergency department.*

**Table 3:** Estimated Cost Savings from Avoided Psychiatric Hospitalizations (2013–2020).

Measure	Value
Total telepsychiatry encounters	19,383
Patients under involuntary commitment (IVC)	13,537
IVCs overturned	4627
Estimated cost per avoided hospitalization	USD 4500
Total estimated cost savings	>USD 20 million
Participating ED sites	53
Uninsured patients (%)	~32

*Cost estimates based on North Carolina “three-way” psychiatric bed reimbursement ($900/day) and a 5-day average inpatient stay.*

**Table 4:** Telepsychiatry Utilization Patterns during COVID-19 by Demographic Factors.

Domain	Key Findings
Overall utilization	Sharp increase during lockdown
Racial differences	Greater increase among White patients
Black patients	Smaller relative utilization increase
Sex differences	Larger proportional increase among women
System performance	High scalability and resilience
Key implication	Persistent equity gaps require targeted strategies

**Table 5:** MOTHeRS Program Outcomes and Reach.

MFM Program Results		
		Program Total to Date
Number pf perinatal patients who received care (visits with MFM specialist)		122
Impact on patient access		36,784 driving miles saved
Number of patient visits with Diabetes Educator or Medical Nutrition Therapist		116
Number of women served for mental health reasons	LCSW visits:	2051
	Psychiatrist visits:	768
	Total Mental Health visits:	2819
Impact on patient access		471,496 driving miles saved
Food Security	Number of Food Boxes sent to Clinics	1195
	Number of Patients Screened for Food Insecurity	41,229
	Number of Food Boxes Distributed	888

*MFM, maternal–fetal medicine.*

**Table 6:** NC-STeP-Peds Program Activities and Outcomes (as of January 2026).

Measure	Value
Children screened	43,858
New psychiatric evaluations	>700
Completed mental health visits	1858
Participating pediatric practices	6
Core care model	Team-based telepsychiatry
Primary benefit	Early identification and access

**Table 7:** University Students’ Mental Health Initiative Outcomes (2022–2025).

Measure	Value
PHQ-9 screenings	2316
GAD-7 screenings	2317
Referrals to counselors	184
Referrals to psychiatrists/NPs	100
Completed clinical sessions	481
Outreach activities	170
Target population	Rural HBCU students

## Data Availability

Data are not publicly available due to institutional restrictions. De-identified, aggregated data may be available upon reasonable request.

## References

[1] Kessler R.C., Chiu W.T., Demler O., Walters E.E. (2005). Prevalence, severity, and comorbidity of twelve-month DSM-IV disorders in the National Comorbidity Survey Replication (NCS-R). Arch. Gen. Psychiatry.

[2] SAMHSA Highlights from 2024 National Survey on Drug Use and Health. https://www.samhsa.gov/data/sites/default/files/NSDUH%202024%20Annual%20Release/2024-nsduh-nnr-highlights.pdf.

[3] Whitney D.G., Peterson M.D. (2019). US national and state-level prevalence of mental health disorders and disparities of mental health care use in children. JAMA Pediatr..

[4] NCDHHS North Carolina Health Professional Shortage Area. 2022 Profile (Current HPSA Data as of 06/01/2023). https://www.ncdhhs.gov/nc-dhhs-orh-hpsa-one-pager/open.

[5] SHEPS Heath Workforce NC. https://nchealthworkforce.unc.edu/interactive/supply.

[6] CDC (2013). Emergency Department Visits by Patients with Mental Health Disorders—North Carolina, 2008–2010. Morb. Mortal. Wkly. Rep. (MMWR).

[7] Saeed S.A., Diamond J., Bloch R.M. (2011). Use of telepsychiatry to improve care for people with mental illness in rural North Carolina. North Carol. Med. J..

[8] Saeed S. (2016). North Carolina Statewide Telepsychiatry Program (NC-STeP): Using Telepsychiatry to Improve Access to Evidence-Based Care. Eur. Psychiatry.

[9] Saeed S.A. (2018). Tower of Babel Problem in Telehealth: Addressing the Health Information Exchange Needs of the North Carolina Statewide Telepsychiatry Program (NC-STeP). Psychiatr. Q..

[10] Saeed S.A. (2018). Successfully Navigating Multiple Electronic Health Records When Using Telepsychiatry: The NC-STeP Experience. Psychiatr. Serv..

[11] Moore B.J., Stocks C., Owens P.L. (2017). Trends in Emergency Department Visits, 2006–2014.

[12] Santo L., Peters Z., DeFrances C.J. (2021). Emergency Department Visits Among Adults with Mental Health Disorders: United States, 2017–2019.

[13] Kothadia R.J., Jones K., Saeed S.A., Torres M.J. (2020). The Impact of the North Carolina Statewide Telepsychiatry Program (NC-STeP) on Patients’ Dispositions From Emergency Departments. Psychiatr. Serv..

[14] Theriault K.M., Rosenheck R.A., Rhee T.G. (2020). Increasing Emergency Department Visits for Mental Health Conditions in the United States. J. Clin. Psychiatry.

[15] Saeed S.A., Jones K., Muppavarapu K. (2022). The Impact of NC Statewide Telepsychiatry Program (NC-STeP) on Cost Savings by Reducing Unnecessary Psychiatric Hospitalizations During a 6½ Year Period. Psychiatr. Q..

[16] NC DHHS Funds for Local Inpatient Psychiatric Beds or Bed Days Purchased in State Fiscal Year 2019–2020 and Other Department Initiatives to Reduce State Psychiatric Hospital Use. https://www.ncdhhs.gov/documents/files/sl-2020-78-section-4e-1-funds-local-inpatient-psychiatric/download.

[17] Xue Y., Saeed S.A., Liang H., Jones K., Muppavarapu K.S. (2022). Investigating the Impact of COVID-19 on Telepsychiatry Use Across Sex and Race: A Study of North Carolina Emergency Departments. Telemed. e-Health.

[18] Saeed S.A., Jones K., Sacks A.J., Craven K., Xue Y. (2023). Maternal Outreach Through Telehealth for Rural Sites: The MOTHeRS Project. North Carol. Med. J..

[19] Coombs N.C., Meriwether W.E., Caringi J., Newcomer S.R. (2021). Barriers to healthcare access among U.S. adults with mental health challenges: A population-based study. SSM-Popul. Health.

[20] Xue Y., Saeed S.A., Muppavarapu K.S., Jones K., Xue L.L. (2023). Exploring the Impact of Education Strategies on Individuals’ Attitude Towards Telemental Health Service: Findings from a Survey Experiment Study. Psychiatr. Q..

[21] Blanco C., Okuda M., Wright C., Hasin D.S., Grant B.F., Liu S.M., Olfson M. (2008). Mental health of college students and their non-college-attending peers: Results from the national epidemiologic study on alcohol and related conditions. Arch. Gen. Psychiatry.

[22] Lipson S.K., Lattie E.G., Eisenberg D. (2019). Increased rates of mental health service utilization by US college students: 10-year population-level trends (2007–2017). Psychiatr. Serv..

[23] Evans T.M., Bira L., Gastelum J.B., Weiss L.T., Vanderford N.L. (2018). Evidence for a mental health crisis in graduate education. Nat. Biotechnol..

[24] Antaramian S. (2015). Assessing psychological symptoms and well-being: Application of a dual-factor mental health model to understand college student performance. J. Psychoeduc. Assess..

[25] Kerr D.C., Capaldi D.M. (2011). Young men’s intimate partner violence and relationship functioning: Long-term outcomes associated with suicide attempt and aggression in adolescence. Psychol. Med..

[26] Fergusson D.M., Boden J.M., Horwood L.J. (2007). Recurrence of major depression in adolescence and early adulthood, and later mental health, educational and economic outcomes. Br. J. Psychiatry.

[27] Summary Report on SFY 2024 North Carolina Statewide Telepsychiatry Program (NC-STeP) Funds, General Statute 143B-139.4B. Report to the Joint Legislative Oversight Committee on Health and Human Services and Fiscal Research Division by North Carolina Department of Health and Human Services November 27, 2024. https://webservices.ncleg.gov/ViewDocSiteFile/104647.

[28] Butzner M., Cuffee Y. (2021). Telehealth Interventions and Outcomes Across Rural Communities in the United States: Narrative Review. J. Med. Internet Res..

[29] Zhang X., Saltman R. (2022). Impact of Electronic Health Record Interoperability on Telehealth Service Outcomes. JMIR Med. Inform..

[30] Wang S., von Huben A., Sivaprakash P.P., Saurman E., Norris S., Wilson A. (2025). Addressing health service equity through telehealth: A systematic review of reviews. Digit. Health.

[31] Petretto D.R., Carrogu G.P., Gaviano L., Berti R., Pinna M., Petretto A.D., Pili R. (2024). Telemedicine, e-Health, and Digital Health Equity: A Scoping Review. Clin. Pract. Epidemiol. Ment. Health.

